# Case Report: Double trouble: a rare case of successfully treated *Mycoplasma hominis* and *Pseudomonas aeruginosa* co-infection

**DOI:** 10.3389/fcimb.2023.1159891

**Published:** 2023-05-01

**Authors:** Shi-Mei Huang, Yu-Rong Tang, Ji-Liang Wang, Xiao-Zhen Wang, Yuan-Yuan Zhang, Su-Fei Pan

**Affiliations:** Department of Clinical Laboratory, Shengli Oilfield Central Hospital, Dongying, Shandong, China

**Keywords:** *Mycoplasma hominis*, *Pseudomonas aeruginosa*, case report, infectious diseases, infection

## Abstract

**Background:**

Extra-urogenital infections due to *Mycoplasma hominis* (*M. hominis*) are rare, particularly co-infection with *Pseudomonas aeruginosa* (*P. aeruginosa*). Herein, we report on a patient who was co-infected and successfully treated despite delayed treatment.

**Case presentation:**

We reported the case of a 43-year-old man with *M. hominis* and *P. aeruginosa* co-infection after a traffic accident. The patient developed a fever and severe infection despite postoperative antimicrobial therapies. The blood culture of wound tissues was positive for *P. aeruginosa.* Meanwhile, culturing of blood and wound samples showed pinpoint-sized colonies on blood agar plates and fried-egg-type colonies on mycoplasma medium, which were identified as *M. hominis* by matrix-assisted laser desorption ionization time-of-flight mass spectrometry (MALDI-TOF MS) and 16S rRNA sequencing. Based on antibiotic susceptibility and symptoms, ceftazidime–avibactam and moxifloxacin were administered for *P. aeruginosa* infection. Meanwhile, after the failure of a series of anti-infective agents, *M. hominis* and *P. aeruginosa* co-infection was successfully treated with a minocycline-based regimen and polymyxin B.

**Conclusion:**

The co-infection with *M. hominis* and *P. aeruginosa* was successfully treated with anti-infective agents despite delayed treatment, providing information for the management of double infection.

## Introduction

*Mycoplasma hominis* (*M. hominis*) commonly colonizes the genitourinary tract in a nonvirulent manner (Whitson et al., 2014). Nevertheless, it is an opportunistic pathogen that can cause a variety of genitourinary or extragenital infections, as well as neonatal infections (Poku, 2022; Zeng et al., 2022). However, this pathogen is commonly underestimated and overlooked in clinical settings due to undetermined pathogenic processes (Ahmed et al., 2021).

Clinically, abscesses, infections of the central nervous system, and bone and joint infections are common extragenital infections, especially in postoperative and immunocompromised patients (Meyer and Clough, 1993; Stabler et al., 2021). Although several extragenital infections due to *M. hominis* have been described, these infections are very rare (Hos et al., 2015), especially bacteremia. For instance, to date, only five cases of *M. hominis* were isolated from the blood over a 10-year period, as reported by the Public Health England reference laboratory (Chalker et al., 2021). Even in China, with the largest population, *M. hominis* was only detected in eight (0.7%) of 1,148 patients with bloodstream infection (Zeng et al., 2022). Due to the limited knowledge on *M. hominis* bacteremia, its clinical features, diagnosis, drug resistance, and treatment recommendations have not yet been fully established.

At present, the diagnosis of invasive *M. hominis* infections remains a clinical challenge owing to the lack of clear symptoms, especially for *M. hominis* bacteremia. As reported by Posse et al. (2018), the conventional method may fail to detect *M. hominis* bacteremia. Meanwhile, empirical antimicrobials generally cannot provide adequate antimicrobial coverage (Wildenbeest et al., 2016); thus, the typical broad-spectrum antibiotic regimens are ineffective for *M. hominis* infections (Whitson et al., 2014), let alone co-infection with another pathogen. Theory predicts increased virulence when multiple strains simultaneously infect the same host, which has major consequences for disease dynamics (Susi et al., 2015), but data on *M. hominis* co-infection are currently scarce. Here, we report on a patient who was co-infected with *M. hominis* and *Pseudomonas aeruginosa* (*P. aeruginosa*) and successfully treated with a minocycline-based regimen and polymyxin B despite delayed treatment.

## Case presentation

**Figure 1:**
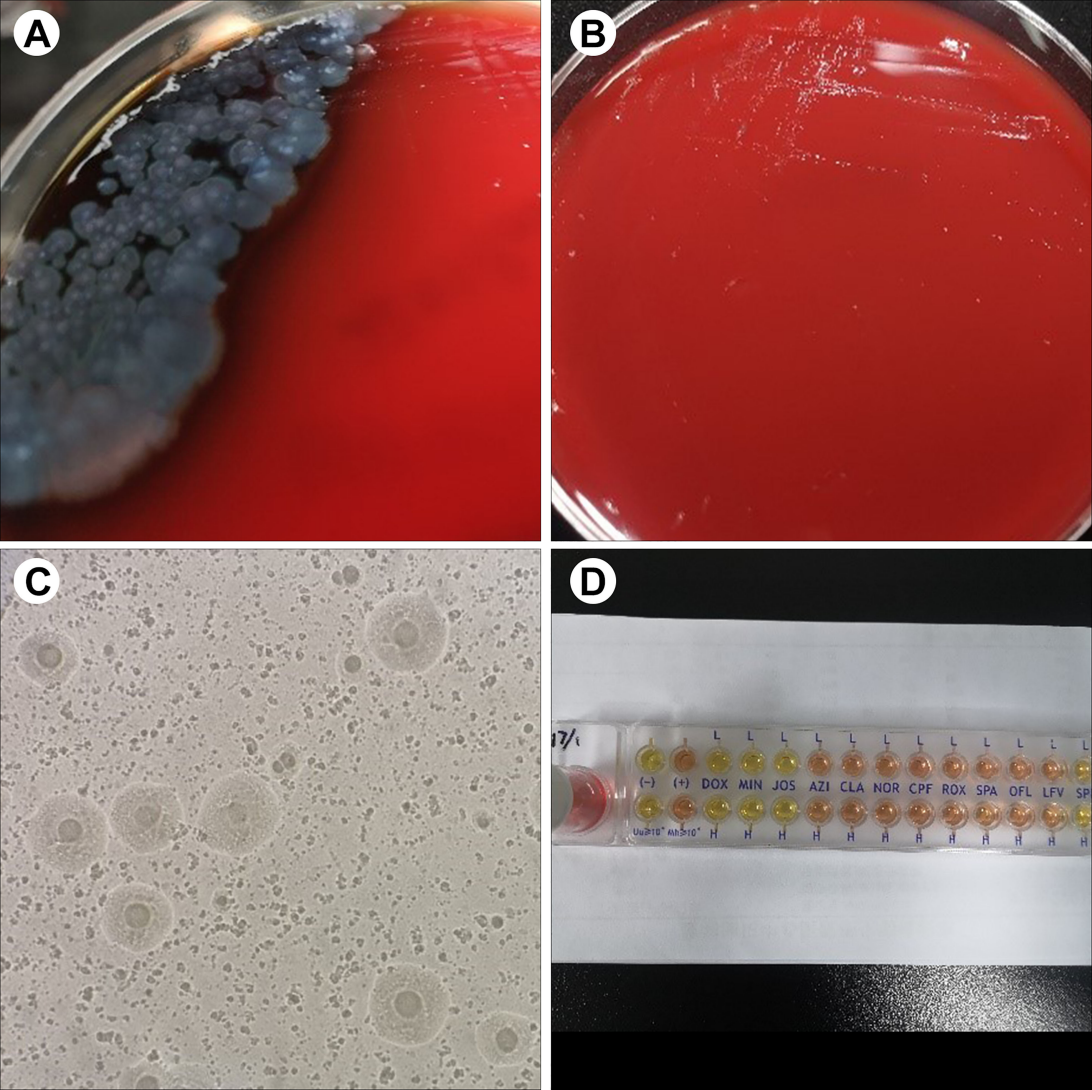
Representative results of sample culture and antibiotic susceptibility testing in patients. **(A)** On day 14, *P. aeruginosa* was positive in the blood culture and bacterial culture of wound tissues. **(B)** On day 28, tiny, nonhemolytic, and transparent colonies were observed on the Columbia blood agar plate. **(C)** Fried-egg-type colonies were observed on mycoplasma medium after 5 days of incubation. **(D)** The results of antibiotic susceptibility testing showed that *M. hominis* was susceptible to doxycycline, minocycline, and josamycin but resistant to azithromycin, clarithromycin, norfloxacin, ciprofloxacin, roxithromycin, sparfloxacin, spectinomycin, and levofloxacin.

A 43-year-old man was admitted to the intensive care unit in our hospital due to multiple open traumas caused by a traffic accident. The patient had a history of hypertension. At admission, the patient presented a constant body temperature of 36.5°C. Physical examination showed no obvious abnormality in the heart, lungs, or abdomen. He suffered multiple lacerations and fractures of the right acetabulum and inferior ramus of the pubis, accompanied by pelvic extraperitoneal hematoma. On the first day of hospitalization, he underwent surgery for multiple injuries. Based on empirical therapy, cefuroxime (1.5 g, every 8 h) was administrated for infection prevention, and anti-infective therapy with cefoperazone–sulbactam (3 g, every 8 h) and levofloxacin (500 mg, daily) was conducted for common bacteria and traumatic wet lung. On day 2, the patient developed a fever (38.4°C), with an increase of procalcitonin (PCT; 31.47 ng/ml) and lactic acid (8.58 mmol/L) after the operation. The antibiotic treatment was changed to meropenem (1.0 g, every 8 h) plus teicoplanin (0.4 g daily). On day 14, *P. aeruginosa* was positive in the bacterial culture of wound tissues (**Figure 1A**) and was identified as a multiple-resistant strain with antibiotic susceptibility testing (AST; Kirby–Bauer method), thus amikacin (0.4 g/day) was added to the anti-infective regimen. Despite antimicrobial therapies, a *P. aeruginosa*-positive blood culture was still identified as bloodstream infection on day 16; the dose of amikacin was therefore adjusted. On day 21, the patient underwent thigh amputation, surgical debridement, and drainage due to recurrent vascular rupture and aggravated cyanosis. Five days later, due to the less effective anti-infective therapy, *P. aeruginosa-*positive blood culture along with fever still existed; therefore, the patient received moxifloxacin to replace the teicoplanin and amikacin. Despite this management, fevers continued, and infection was still under suspicion. Ceftazidime–avibactam (2.5 g/day) was then administrated to replace the meropenem. The culture was *P. aeruginosa* negative for the 26th-day blood sample but still positive for wound tissues.

**Figure 2:**
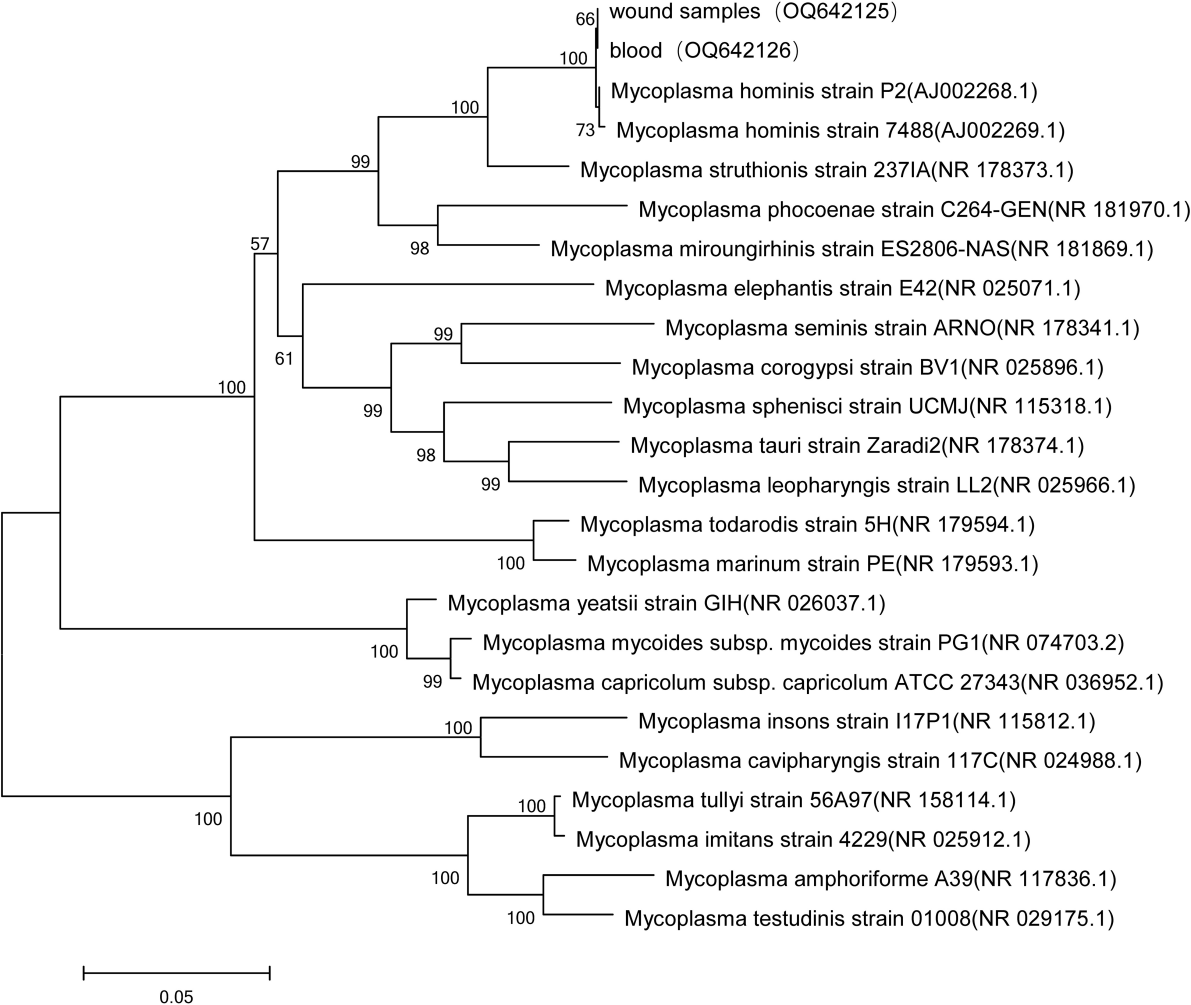
Phylogenetic analyses of *M. hominis* isolated from wound tissue and blood samples.

**Figure 3:**
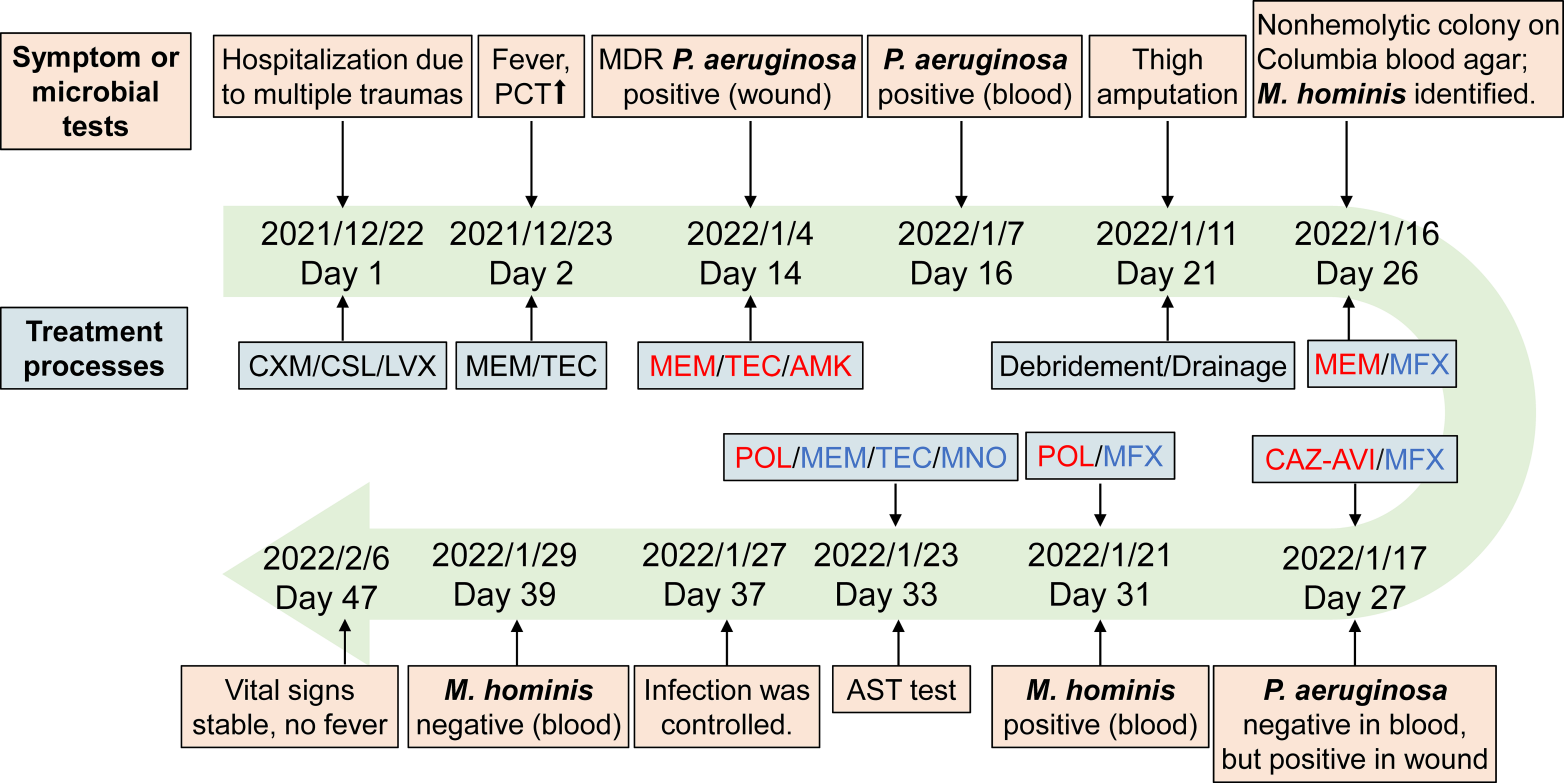
Timelines of patient’s diagnosis and treatment. Red, the anti-infective drugs for *P. aeruginosa*. Blue, the anti-infective drugs for *M. hominis*. Abbreviations: CXM, cefuroxime; CSL, cefoperazone-sulbactam; LVX, levofloxacin; MEM, meropenem; TEC, teicoplanin; AMK, amikacin; MFX, moxifloxacin; CAZ-AVI, ceftazidime–avibactam; POL, polymyxin B; MNO, minocycline.

Due to the uncontrolled infection, other pathogens were suspected. On day 26, tiny, nonhemolytic, and transparent colonies grew on the Columbia blood agar plate of four blood sample cultures (**Figure 1B**), possibly representing *M. hominis.* Gram staining of the blood smear showed no bacteria. A subculture of blood and wound tissue samples on mycoplasma medium presents as fried-egg-type colonies after 5 days of incubation (**Figure 1C**). Colonies were then identified to be *M. hominis* by the matrix-assisted laser desorption ionization time-of-flight mass spectrometry (MALDI-TOF MS) and further confirmed by 16S rRNA sequencing (primers: 27F, AGAGTTTGATCMTGGCTCAG; 1492R, GGTTACCTTGTTACGACTT) and phylogenetic tree analysis (**Figure 2**; GenBank Accession No. OQ642125 for a strain isolated from a wound tissue sample and OQ642126 for a strain isolated from a blood sample). On day 31, therapy with polymyxin B in a dose of 5 × 10^5^ U/day was initiated instead of ceftazidime–avibactam due to its shortage. In addition to the initial isolates of *M hominis*, two subsequent cultures obtained in the following week were also positive. The AST with a commercial kit (broth dilution method, Zhongaisheng, Hebei, China) showed that *M. hominis* was susceptible to doxycycline, minocycline, and josamycin but resistant to azithromycin, clarithromycin, norfloxacin, ciprofloxacin, roxithromycin, sparfloxacin, spectinomycin, and levofloxacin (**Figure 1D**). Based on the results of AST, minocycline (100 mg, twice/day), meropenem plus teicoplanin were started instead of moxifloxacin on the 33rd day of hospitalization. On the 37th hospital day, the hematology data and PCT level returned to normal, and infection was controlled. After starting minocycline-based therapy for 6 days, repeated cultures from the blood were *M. hominis* negative. Details regarding the diagnosis and treatment are shown in **Figure 3**.

## Discussion and conclusions

Accumulating evidence has demonstrated that co-infection by multiple pathogen strains is a double trouble for global public health, which increases the risk of disease severity as well as the difficulty in clinical diagnosis and treatment (Kehr and Engelmann, 2015; Zhu et al., 2020). In this case report, we report on a patient who experienced double trouble, that is, *M. hominis* and *P. aeruginosa* co-infection. This co-infection was successfully treated with anti-infective agents despite delayed treatment.

Bloodstream infection due to *M. hominis* is rarely reported (Wang et al., 2022), let alone co-infection with another pathogen. In this report, the patient was co-infected with *M. hominis* and *P. aeruginosa*, which manifested as fever and an increase of PCT and lactic acid. These clinical manifestations are in accordance with the reported typical symptoms of *M. hominis* bloodstream infection (Zeng et al., 2022). *P. aeruginosa* blood infection was successfully treated with short-term antibiotics therapy. However, similar to previous cases (Henao-Martínez et al., 2012; Pailhoriès et al., 2014), the patient showed poor response to the wide antimicrobial treatments and developed persistent fever due to *M. hominis* infection. Additionally, delayed wound closure, another clinical manifestation of *M. hominis* infection, was also observed in our case, which was similar to the report by Adams et al. (2020). Thus, the identification of *M. hominis* should be considered in multiple-trauma patients who developed unexplained postoperative fever and protracted wound healing, particularly those who have a poor response to wide-spectrum antibiotics.

To date, the identification of *M. hominis* is challenging under conventional techniques (e.g., direct examination and culture of specimens), leading to the underestimation of this pathogen. Due to the absence of a cell wall, *M. hominis*, as an atypical bacteria, cannot be detected by Gram staining of clinical specimens. Conversely, in our case, *P. aeruginosa* infection was identified easily by Gram staining due to definite colony morphology, manifesting as yellow–green colonies. Accordingly, the *P. aeruginosa* infection was first treated appropriately. However, due to the uncontrolled infection, other pathogens were suspected. By the prolonged incubation (~5 days) on blood agar, tiny, nonhemolytic, and transparent colonies were observed, which is consistent with the previously reported colony morphology (Xiang and Lu, 2019; Wang et al., 2022). At this point, we need to remind readers that prolonged incubation is necessary to allow *M. hominis* colonies to develop, and in addition, translucent *M. hominis* colonies may be overlooked and should be examined carefully under reflected light. As recommended by Stabler et al. (2021), *M. hominis* should be suspected when Gram staining failed to detect microorganisms from pinpoint-sized, transparent colonies, warranting subculture onto mycoplasma medium. Previously, 16S rRNA sequencing and MALDI-TOF MS have been proven to be very useful for the rapid identification of *M. hominis* (Pereyre et al., 2013; Pailhoriès et al., 2014). Thus, with the suspicion of M. *hominis*, the colonies isolated from our case were sent to be identified by 16S rRNA sequencing and MALDI-TOF MS in order to prove the presence of microorganisms. As previously reported, a score of ≥ 1.70 rather than the classical threshold of ≥ 2.00 for Bruker MALDI-TOF MS was enough for accurate species-level identification of *M. hominis* (Pereyre et al., 2013); in our case, a score of 1.902 was achievable after 5 days of incubation on blood agar, supporting this result. Overall, *M. hominis* was definitely identified, which also supported the feasibility of MALDI-TOF MS or molecular methods in its rapid identification. Thus, for the rapid identification of this pathogen, we recommend that MALDI-TOF MS be necessary once colonies are isolated.

Empirical therapy for *M. hominis* infection includes the typical broad-spectrum antibiotic regimens (e.g., beta-lactam antimicrobials, vancomycin); however, these agents are generally ineffective since they act on cell wall metabolism (Yamaguchi et al., 2009; Bergin et al., 2017). So far, there has been no consensus on the treatment of *M. hominis* infection. In previously published cases, therapy/treatment options included drainage, debridement, and specific antibiotic therapy (Zhou et al., 2016). In our case, the patient underwent drainage and debridement because of ineffective anti-infective therapy. Thus, surgery may be considered a promising treatment option and should be performed promptly, as Yamaguchi et al. (2009) recommended. Several anti-infective agents, such as doxycycline (Henao-Martínez et al., 2012; Pailhoriès et al., 2014) and imipenem (Cuchý et al., 2000), have been used successfully in some cases of *M. hominis* infection; nevertheless, there is a lack of consensus about their efficacy. There are also conflicting views pointing out that specific antibiotic therapy for *M. hominis* was unnecessary (Cuchý et al., 2000). Even so, the present study supported that specific antibiotic therapy is preferable for the *M. hominis* infection since our patient was finally cured using antimicrobials. Despite the patient having been treated with various wide-spectrum antibiotics, he developed a persistent fever but excitingly recovered after adding minocycline, which was consistent with previous experience (Zhou et al., 2016). Actually, existing evidence has already indicated that tetracycline was considered to be the drug of choice for *M. hominis* infections (Myhre and Mårdh, 1983). On the basis of our report and existing evidence, tetracycline might be an effective option for *M. hominis* infection if the strain is not resistant.

Nevertheless, currently, the use of antimicrobials has led to a rapid increase in the emergence of resistant *M. hominis* strains (Cummings and McCormack, 1990; Pereyre et al., 2002; Meygret et al., 2018). For example, *M. hominis* are innately resistant to all agents acting on bacterial cell wall replication (e.g., β-lactams, sulfonamides, trimethoprim, rifampin) or folic acid synthesis (e.g., sulfonamides) (McCormack, 1993; Ahmed et al., 2021). The *M. hominis* isolates in our report were susceptible to doxycycline, minocycline, and coxacycline but are intrinsically resistant to a variety of antibiotic classes, such as azithromycin, roxithromycin, and levofloxacin. This antibiotic susceptibility profile is generally similar to the case reported by Reissier et al. (2016) and in line with the commonly reported antimicrobial susceptibility patterns of *M. hominis* (Kenny and Cartwright, 2001; Redelinghuys et al., 2014). Thereby, considering that the antibiotic susceptibility profile of each isolate varied irregularly (Cuchý et al., 2000; Pailhoriès et al., 2014), the appropriate AST testing for antimicrobial agents suitable for *M. hominis* infections, including macrolides, lincosamides, streptogramins, tetracyclines, and fluoroquinolones, should be applied before antibiotic therapy. Nevertheless, empirical therapy is necessary and required before antimicrobial test results.

In conclusion, extra-urogenital infections due to *M. hominis* are rare, especially co-infected with another pathogen. Despite the increased risk of disease severity and difficulty in treatment, the *M. hominis* and *P. aeruginosa* co-infection are treatable with appropriate antimicrobial agents. Moreover, this case highlighted the pathogenic potential of *M. hominis* in the bloodstream. *M. hominis* bloodstream infection is recommended to be considered in multiple-trauma patients with unexplained fever, particularly in the absence of response to wide-spectrum antibiotics.

## Data availability statement

The original contributions presented in the study are included in the article/supplementary material. Further inquiries can be directed to the corresponding author.

## Ethics statement

The studies involving human participants were reviewed and approved by Ethics committee of Shengli Oilfield Central Hospital. The patients/participants provided their written informed consent to participate in this study. Written informed consent was obtained from the participant/patient(s) for the publication of this case report.

## Author contributions

Conception and design: S-MH and J-LW. Provision of study materials or patients: Y-RT and S-FP. Data analysis and interpretation: X-ZW and Y-YZ. Administrative support: S-FP. Manuscript writing and reviewing: all authors. All authors contributed to the article and approved the submitted version.
